# Investigating a cholera outbreak in Kaiso Fishing Village, Hoima District, Uganda, October 2015

**DOI:** 10.11604/pamj.supp.2018.30.1.15281

**Published:** 2018-05-18

**Authors:** Monica Okuga, David Were Oguttu, Allen Eva Okullo, Meeyoung Mattie Park, Charles Perry Ko, Joseph Asamoah Frimpong, Bao-Ping Zhu, Alex Riolexus Ario

**Affiliations:** 1 Makerere University School of Public Health, Kampala, Uganda; 2 Makerere University Centre for Maternal, Newborn and Child Health, Kampala, Uganda; 3 Uganda Public Health Fellowship Program, Kampala, Uganda; 4 Rollins School of Public Health, Emory University, Atlanta, USA; 5 African Field Epidemiology Network, Accra, Ghana; 6 US Centres for Disease Control and Prevention, Kampala, Uganda; 7 Ministry of Health, Kampala, Uganda

**Keywords:** Outbreak investigation, cholera, Uganda

## Abstract

Globally, even though improvements have been made to effective surveillance and response, communicable diseases such as cholera remain high priorities for national health programs, especially in Africa. High-quality surveillance information coupled with adequate laboratory facilities are effective in curbing outbreaks from such diseases, ultimately reducing morbidity and mortality. One way of building this capacity is through simulation of response to such health events. This case study based on a cholera outbreak investigated by FETP trainees in October 2015 in Uganda can be used to reinforce skills of frontline FETP trainees and other novice public health practitioners through a practical simulation approach. This activity should be completed in 2.5 hours.

## How to use this Study

**General instructions:** this exercise is led by 1-2 facilitators with a class size of 8-15 students. It is participatory and the facilitator should encourage all students to take part through reading out aloud, attempting the questions, sharing answers aloud, giving opinions and critiquing each other. In some cases, role plays may be performed.

**Audience:** District Surveillance officers, Field Epidemiology Training Program (FETP) trainees.

**Prerequisites:** before using this case study, case study participants should have received lectures in outbreak investigation and basic biostatistics. They should also be proficient in English and conversant with Microsoft Excel.

**Materials needed:** laptop with Microsoft Excel, flipchart/white board, markers.

**Level of training and associated public health activity**: Novice – outbreak investigation

**Time required**: 2.5 hours

**Language**: English

## Competing interest

The authors declare no competing interest.
